# Nanodomain structure of single crystalline nickel oxide

**DOI:** 10.1038/s41598-021-82070-1

**Published:** 2021-02-10

**Authors:** B. Walls, A. A. Mazilkin, B. O. Mukhamedov, A. Ionov, I. A. Smirnova, A. V. Ponomareva, K. Fleischer, N. A. Kozlovskaya, D. A. Shulyatev, I. A. Abrikosov, I. V. Shvets, S. I. Bozhko

**Affiliations:** 1 School of Physics and Centre for Research on Adaptive Nanostructures and Nanodevices (CRANN), Trinity College Dublin, Dublin 2, Ireland; 2 Institute of Solid State Physics, Russian Academy of Sciences, Chernogolovka, Russia; 3 Materials Modeling and Development Laboratory, NUST MISIS, Leninskiy prosp, 4, Moscow, Russia 199049; 4 School of Physical Sciences, Dublin City University, Dublin 9, Ireland; 5 Department of Physics, Chemistry and Biology (IFM), Linköping University, 58183 Linköping, Sweden

**Keywords:** Nanoscience and technology, Physics, Condensed-matter physics, Structural properties, Materials science, Condensed-matter physics, Structure of solids and liquids

## Abstract

In this work we present a comprehensive study of the domain structure of a nickel oxide single crystal grown by floating zone melting and suggest a correlation between point defects and the observed domain structure. The properties and structure of domains dictate the dynamics of resistive switching, water splitting and gas sensing, to name but a few. Investigating the correlation between point defects and domain structure can provide a deeper understanding of their formation and structure, which potentially allows one to tailor domain structure and the dynamics of the aforementioned applications. A range of inhomogeneities are observed by diffraction and microscopy techniques. X-ray and low-energy electron diffraction reveal domains on the submicron- and nanometer-scales, respectively. In turn, these domains are visualised by atomic force and scanning tunneling microscopy (STM), respectively. A comprehensive transmission electron microscopy (TEM) study reveals inhomogeneities ranging from domains of varying size, misorientation of domains, variation of the lattice constant and bending of lattice planes. X-ray photoelectron spectroscopy and electron energy-loss spectroscopy indicate the crystal is Ni deficient. Density functional theory calculations—considering the spatial and electronic disturbance induced by the favourable nickel vacancy—reveal a nanoscale distortion comparable to STM and TEM observations. The different inhomogeneities are understood in terms of the structural relaxation induced by ordering of nickel vacancies, which is predicted to be favourable.

## Introduction

Metal oxides display a wide variety of physical properties, stimulating interest from the point of view of fundamental physics and device engineering. NiO is antiferromagnetic and a charge transfer insulator. However, nickel vacancies are common and give rise to an effective p-type doping^1^. NiO sees application in spin valves^2^, supercapacitors^3^, hole transport layer in perovskite solar cells^4^, water splitting (Fe doped NiO)^5^ and gas sensing^6^.

Floating zone melting (FZM) is one of the most suitable techniques for the growth of high quality metal oxide single crystals (for detailed review see^7,8^). Despite the high quality of single crystals grown by FZM, different types of structural and chemical inhomogeneities can exist. Crystal lattice defects in FZM single crystals such as domain boundaries, dislocation walls, twins and stacking faults affect the structure of X-ray diffraction (XRD) reflexes^9^. Lab based X-ray sources can probe the coherent domain size and overall crystalline quality^10,11^ but cannot easily distinguish between or study particular two-dimensional defects. The standard statistical approach of X-ray diffractometry allows one to obtain information averaged over ensembles in the kinematical^12,13^, semi-dynamical^14^ or dynamical approaches^15^. However, comprehensive investigation of crystal structure requires the combination of methods providing statistically averaged information and high spatial resolution electron and scanning probe microscopy techniques. Point defects are considered to be scattered homogeneously throughout the crystal and appear in diffraction spectra as a background signal with the reflex structure unaffected. Consequently, even high-quality single crystals, as judged by diffraction techniques, can contain a relatively high concentration of point defects.

The macroscopic properties of most metal oxides are extremely sensitive to the presence of point defects. Focusing on monoxides, cation vacancies can induce fascinating properties such as ferromagnetism in the case of CoO^16^ and half-metallicity in the case of MnO^17^, CoO^18^ and NiO^17^. In the latter, single defects can produce half-metallicity in either spin channel locally. However, the interaction of vacancies resulting in half-metallicity in the same spin channel is predicted to be energetically favourable^19^. Nickel oxide’s antiferromagentic order is predicted to persist even in the presence of the cation vacancy and resulting half-metallicity^17^. The resistive switching observed in a variety of metal oxides including NiO^20–23^ appears to be due to intrinsic point defects. These defects produce states at various levels within the band gap, which are likely responsible for transport within the insulator.

Larger-scale structural inhomogeneities are known to strongly influence physical phenomena and applications. For example, electric field induced conductive filaments in NiO films form predominately at domain boundaries^19,24,25^. Therefore, boundary density and structure dictate the resistive switching properties. The gas sensing and water splitting performance of metal oxides is also strongly correlated to domain size^26–28^.

Therefore, studying domain structure and how vacancies can dictate domain structure is vital. In this work we reveal a complex domain structure of a NiO single crystal and demonstrate that it can be correlated to point defects. Diffraction techniques reveal both submicron- and nanometer-scale domains, which are visualised by microscopy techniques. Electron energy-loss spectroscopy (EELS) and X-ray photoelectron spectroscopy (XPS) reveal a Ni deficiency and density functional theory (DFT) calculations demonstrate the Ni vacancy induces a nanometer-scale distortion. Analysis of these measurements and calculations allow us to propose a correlation between the point defects and the domains of different size.

## Results

### Experimental results

**Figure 1:**
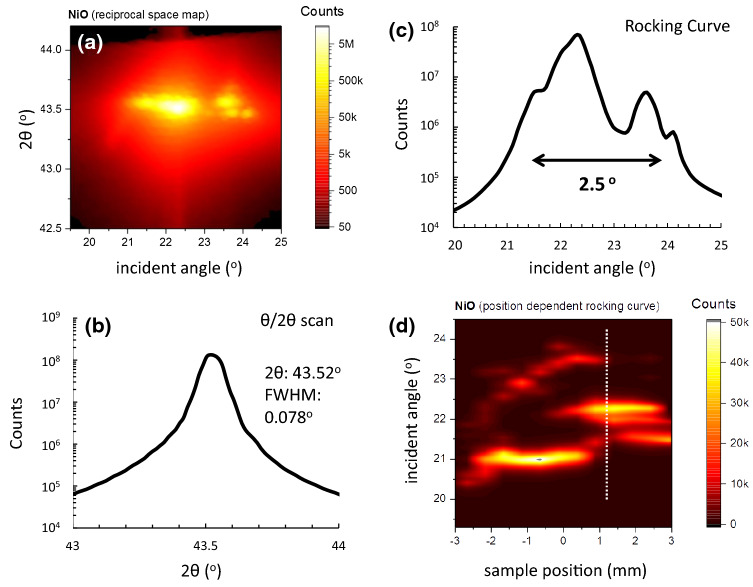
(**a**) Reciprocal space map shown in terms of incident angle and 2$\theta$ angle. (**b**) shows the $\theta$/2$\theta$ scan for the NiO single crystal obtained by summing all the columns of the reciprocal space map. The average coherent domain size is estimated to be 115 ± 2 nm. (**c**) shows the corresponding rocking curve derived from summing all the rows of the reciprocal space map. The indicated angular interval shows that there are misoriented areas within the probed sample volume. (**d**) Rocking curves as a function of sample position. The sample was moved by 0.5 mm per step. The misorientation of the domain is maintained over the probed region. The dotted line indicates the region of the sample where (**a**–**c**) have been taken.

The domain structure of the FZM grown NiO single crystal has been examined in detail by XRD measurements of the [002] reflex^29^, depicted in Fig. 1. The lattice constant is estimated to be 4.154 ± 0.004 Å, comparable to the reported value of 4.17Å^30,31^. The reciprocal space map (RSM) is depicted in Fig. 1a. Figure 1b shows the $\theta$/2$\theta$ scan obtained by summing all the columns of the RSM. The misorientation of local regions of the crystal leads to a broadening of the reflex in 2$\theta$. This is due to the mosaic structure of the crystal and the deformation of the crystal locally. Using the Scherrer equation we can estimate the average coherent domain size to be 115 ± 2 nm. The corresponding rocking curves, derived by summing all the rows of the RSM, is presented in Fig. 1c. The analysed spot size is 1 $\times$ 5 mm and within the XRD scattering volume we see two distinct reflexes separated by an angular offset of around 2$^{\circ }$. We have to stress that in symmetric XRD scans we are not directly sensitive to the lateral domain size but rather towards the columnar structure normal to the crystal surface. Taking rocking curves at several areas of the sample in 0.5 mm steps (see Fig. 1d) reveals that the tilt of the domains is maintained over the probed region. Please note that due to the line focus we can only map in one direction. Intensities are also not directly proportional to the volume of the domains as angular optimisation in the second tilt direction was only performed on one specific misorientation.

**Figure 2:**
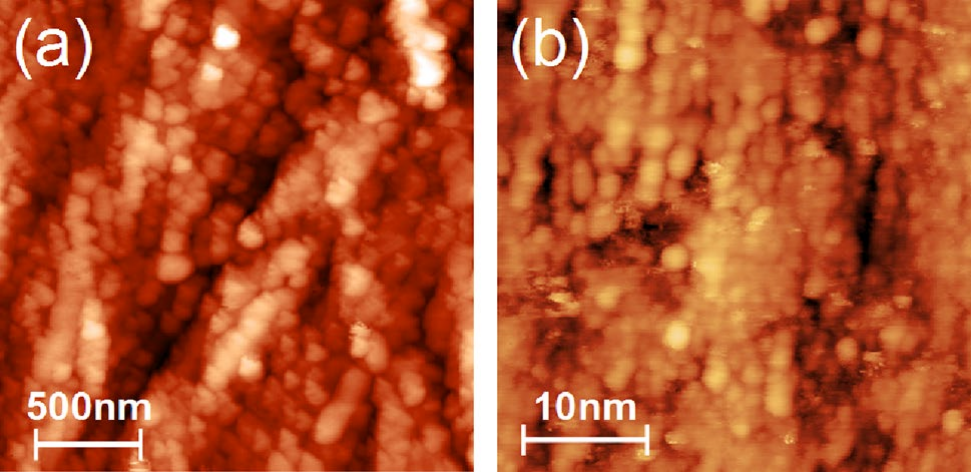
(**a**) AFM image (2.5 $\times$ 2.5 $\upmu$m$^2$) of the NiO(001) surface and (**b**) STM image (30 $\times$ 30 nm$^2$) of the Ni$_{1-x}$Li$_x$O (001) surface. The domains are around 100 nm and several nanometers in size in AFM and STM images, respectively.

A tapping mode atomic force microscopy (AFM) measurement of the crystal is presented in Fig. 2a. Features of around 100 nm are observed, consistent with the coherent domain size observed in XRD. Clusters of spherical domains have been observed on cobalt doped NiO single crystalline films^32^: the average particle size was found to vary between 108 and 156 nm, comparable to this work.

In Fig. 2b a scanning tunneling microscope (STM) image of a Li doped NiO single crystal is presented. The measurement has been performed on a doped crystal as the undoped crystal was not sufficiently conductive for STM measurements. The doped crystal has been grown by the same method as the updoped crystal and XPS measurements estimate a Li dopant concentration of 0.4 ± 0.1% (See Supplementary Fig. S3 online). Similarly to the undoped crystal, the Ni$_{1-x}$Li$_x$O single crystal’s domain structure has also been examined in detail by XRD: it is comparable to the undoped crystal and is discussed in the Supplementary Information (Supplementary Fig. S1 online). Prior to the constant-current mode STM measurement the doped crystal has been subjected to multiple sputter anneal cycles (see Methods section below). The STM image demonstrates that in addition to the submicron-scale domains observed by XRD, nanodomains are present. The nanodomains have a typical size of a few nanometers. The local corrugation between adjacent nanodomains of up to a nanometer cannot be purely an electronic effect, and hence, the contrast reflects the topography of the surface. This nanodomain structure could not be removed or changed by ion etching and annealing in ultra-high vacuum, which indicates the structure is not due to contamination or random disorder but is inherent to the bulk of the crystal.

**Figure 3:**
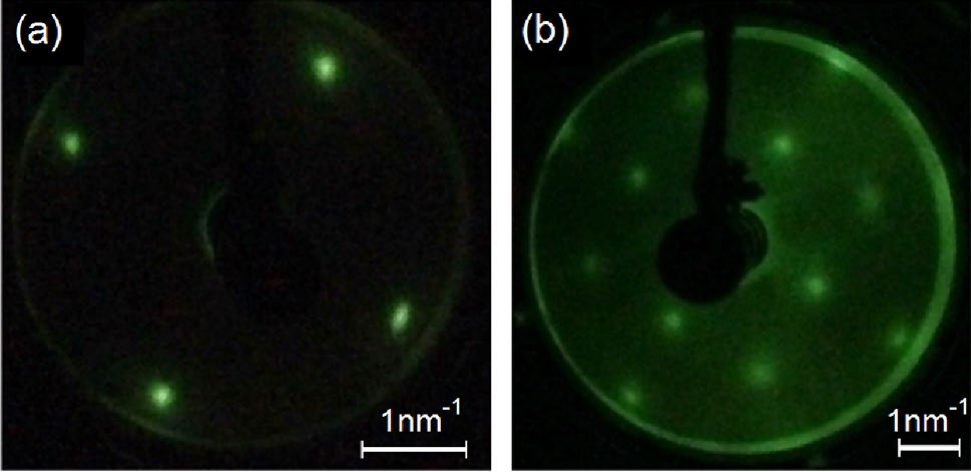
LEED of (**a**) Ni$_{1-x}$Li$_x$O(001) and (**b**) NiO(001) surfaces after ion etching and annealing. (**a**) $\hbox {E}=45\,\hbox {eV}$ and (**b**) $\hbox {E}=115\,\hbox {eV}$. From the width of the spot profile in (**a**) the coherent area size is estimated to be $d=6$–8 nm.

A low-energy electron diffraction (LEED) measurement of the doped crystal—obtained immediately after the STM measurement—is depicted in Fig. 3a. The undoped crystal has been subjected to the identical sputter-anneal treatment and its LEED pattern is presented in Fig. 3b. The cubic reciprocal space unit cells correspond to the (001) surface of NiO. In both cases the lattice constant is estimated to be 4.3 ± 0.2 Å. Note the quality of the diffraction patterns after ion etching and annealing, illustrating the crystallographic correlation of the nanodomains. Estimation of the coherent area size from the width of the LEED spots gives a value of 6–8 nm for the doped and undoped crystals. The nanodomain structure of the undoped NiO crystal is examined in detail by TEM below. However, the STM and LEED measurements suggests nanodomains exist at the surface. This is critical to gas sensing and water splitting^26–28^.

**Figure 4:**
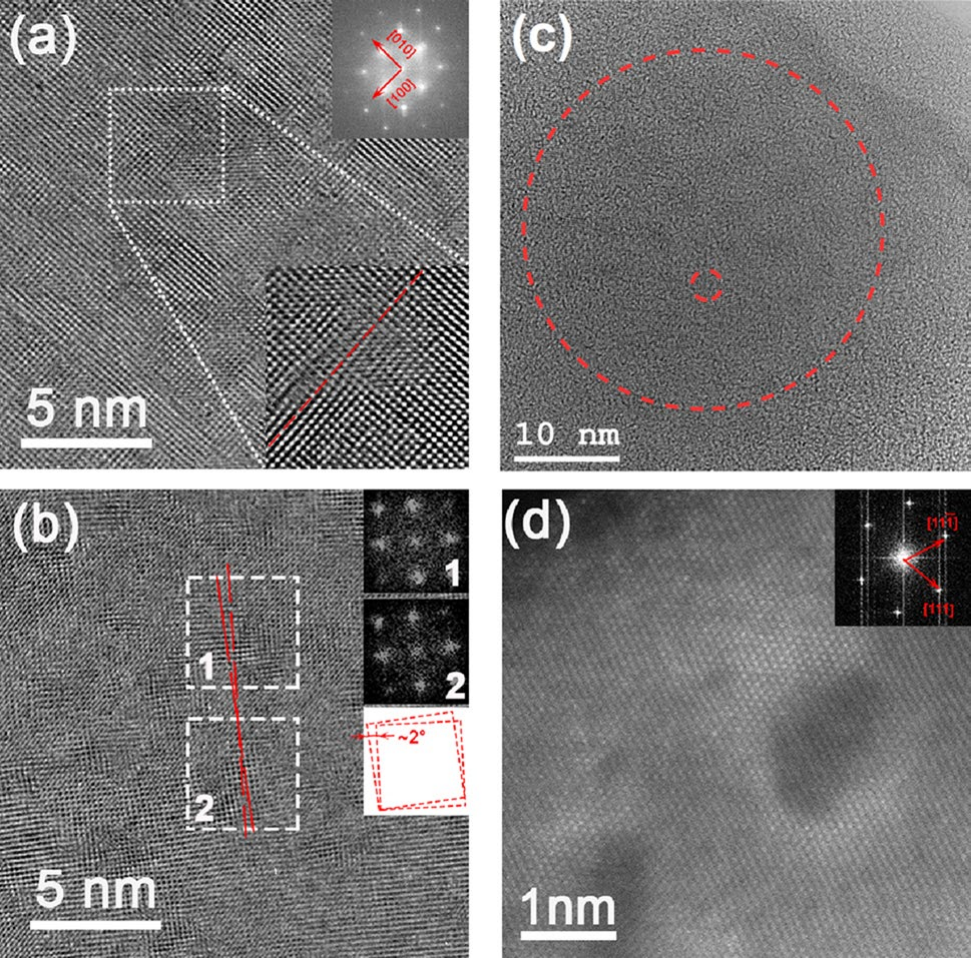
(**a**–**c**) HR TEM image of the NiO single crystal; sample normal is parallel to the [001] direction. (**a**) Structure contains linear features in two perpendicular directions marked by arrows. Reflection blurring in the corresponding FFT (inset top) can indicate the domain structure of the NiO crystal. The FFT filtered portion of the image is enlarged in the bottom right inset. The bending of a lattice fringe is highlighted by the dashed red line, which serves as an eye-guide. (**b**) Small scale HR TEM image reveals the presence of the crystalline domains. Two FFTs from neighbouring areas 1 and 2 demonstrate in-plane lattice rotation of approximately 2$^{\circ }$ that is shown schematically by two red misoriented squares. Two red dashed lines in (**b**) serve as an eye-guide to demonstrate the in-plane domain rotation. Disturbance of atomic resolution contrast confirms the out-of-plane lattice rotation. (**c**) The small dashed circle highlights an example of a small domain, similar in size to those visualised in (**a**), which agglomerate to form a larger feature (large circle). (**d**) HR STEM HAADF image. Electron beam is parallel to [110] NiO direction. Contrast indicates regions attributed to different nickel density.

Figure 4a–c depicts high-resolution transmission electron microscopy (HR TEM) images of the NiO crystal with the electron beam parallel to [001] NiO direction. TEM measurements estimate the lattice constant to be 4.08 ± 0.05 Å. A specific feature of the TEM images is the non-uniformity of the contrast. Linear features along the perpendicular [100] and [010] crystallographic directions in Fig. 4a are evident. The image indicates that the NiO crystal is split into $\sim$ 2–3 nm cells. Some of these cells are perfectly oriented along the [001] zone axis showing atomically resolved contrast, while the neighbouring cells are misaligned with lattice fringes visible only in the [100] or [010] direction. The presence of the misoriented areas is also supported by the fast Fourier transform (FFT), where the blurring of the lattice reflections is observed. This nanoscale inhomogeneity justifies the domain structure revealed by STM. The enlarged portion of the HR TEM image (Fig. 4a bottom inset) also reveals the bending of a lattice fringe highlighted by the dashed red line; atomic columns are shifted in the middle part of the line. The presence of nano-scale cells in the NiO single crystalline sample is also shown in Fig. 4b. The two FFTs, which both correspond to the (002) plane, reveal the two regions are (001) orientated. However, the orientation of the local (200) lattice fringes differ. This is highlighted by the two non-parallel red lines and the comparison of the FFTs. Hence, the cells are aligned with respect to the surface normal but misaligned in the surface plane by an angle of around 2$^{\circ }$ (see the schematics in the insert). The disturbance of atomic resolution contrast in particular areas confirms the out-of-plane lattice rotation. The few degrees misorientation of the NiO domains observed by HR TEM is in accordance with the XRD measurements. In Fig. 4c the submicron-scale domain (big circle) appears to be an aggregation of nanodomains (small circle). The size of the former and the latter are on the same scale as the features observed in AFM and STM measurements, respectively. This infers a correlation between the domains observed on the nano- and submicron-scale.

We also performed TEM measurements with the electron beam parallel to [110] NiO direction. Figure 4d is the STEM high angle annular dark field (HAADF) image. Here we observe areas with darker contrast, both equiaxial and elongated, from one to several nanometers in size. In the case of elongated areas, their larger axis lies close to the [100] crystallographic direction. From this image, we can conclude that these nanodomains are located in the (001) plane of the NiO crystal. Usually the contrast in a STEM HAADF image is correlated to the atomic number of the elements by the power law, $\sim$ Z$^{1.8}$ (Z-contrast). Therefore, the contrast in Fig. 4d is correlated to the density of Ni ions, while the oxygen is not visible due to its lower atomic number. However, we should note that HR STEM is more sensitive to local sample orientation than HR TEM. As a result, we can interpret the low-contrast areas in Fig. 4d as nanodomains misoriented with respect to the adjacent area. In this case, when the local orientation is deviated from the zone axis, it is difficult to separate domain misorientation from Ni content. Although one should note that nickel content and misorientation can be inherently linked.

EELS estimation of the NiO elemental composition—depicted in the Supplementary Information (Supplementary Fig. S2 online)—demonstrates a small deficiency of Ni cations. EELS measurements were performed on multiple locations (5 different samples and around 10 measurements for each sample), and every time we calculated a Ni deficiency. Statistically the Ni deficit was about 2% (48:52). XPS measurements (Supplementary Fig. S3 online) of the sputtered surface shows only the presence of Ni and O peaks with very small peaks attributed to argon and carbon. The atomic ratio of Ni to O was estimated to be 48:52 (2% deficit of Ni). The accuracy of XPS quantification is about 1–1.5% (details are presented in the Supplementary Information). To preserve the overall electrical neutrality in a NiO crystal with a Ni deficit, some Ni$^{2+}$ is converted to Ni$^{3+}$. The Ni$^{3+}$ ions were also detected by XPS. The observation of nickel deficiency is not surprising; NiO is a p-type semiconductor in normal growth conditions^1^. In equilibrium defect chemistry, NiO usually has a nickel deficiency accommodated by nickel vacancies^33,34^. The Ni vacancy formation energy is low in comparison with the O vacancy formation energy^35^. Due to the relative error of both the EELS and XPS measurements we cannot quantitatively determine the Ni content. However, considering the XPS and EELS measurements and the above considerations we strongly suggest the NiO crystal is Ni deficient and this deficiency is due to nickel vacancies. The non-stoichiometry indicates the point defects and the inhomogeneity they create locally may be correlated to the observed domain structure. In order to investigate this we have performed DFT calculations examining the spatial and electronic disturbance of a nickel vacancy.

### Density functional theory calculations

One way to treat systems with strong correlations like NiO is an implementation of a Hubbard Coulomb term U and exchange parameter J into DFT framework^36^. We have tested the GGA+U method with different values of $U_{\text {eff}}$ ($U_{eff}=U-J$) and compared the calculated band gap, lattice parameter and magnetic moment with experiment. Table 1 compares the calculated properties with available experimental data for $U=9\,\hbox {eV}$ and $J=1\,\hbox {eV}$, i.e. $U_{eff}=8\,\hbox {eV}$. Our theoretical results on the lattice parameter, on-site magnetic moment and band gap agree well with experimental data from^30,37–40^, which provides confidence in the chosen values of *U* and *J* parameters for the Ni atoms. The predicted lattice parameter is 4.17Å. For comparison, in this work we estimate the lattice constant to be $4.154\pm 0.004$ Å, $4.08\pm 0.05$ Å and $4.3\pm 0.2$ Å by XRD, TEM and LEED, respectively.

**Table 1:** Calculated properties of bulk NiO and available experimental data.

Property	Theory (this work)	Experiment
Lattice parameter, Å	4.17	4.17^30,37^
Magnetic moment, $\mu _b$	1.815	1.9^30^, 1.77^38^
Band gap, eV	4.0	4.2^37^, 4.3^39^, 4.0^40^

**Figure 5:**
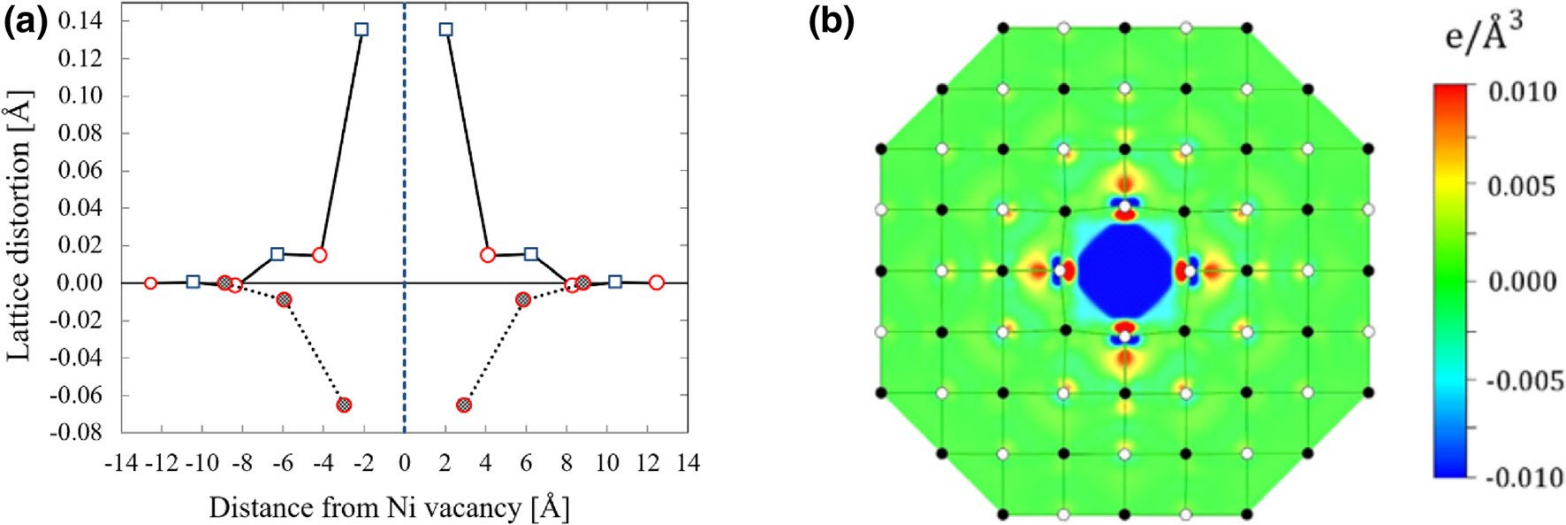
(**a**) Lattice distortion induced by a charged Ni vacancy (V$_{\text {Ni}^{2-}}$). Distortions along [100] and [110] crystallographic axes are plotted with solid and dotted lines, respectively. Circles and squares denote Ni and O atoms, respectively. Positive and negative values of the lattice distortions indicate an outward and inward deformations, respectively. (**b**) Charge density difference induced by the Ni vacancy (V$_{\text {Ni}^{2-}}$) in the (001) plane. Negative (blue colour) and positive (red colour) corresponds to a reduction or an increase of the charge density compared to the bulk cell, respectively.

For simulating the effects from a Ni vacancy, we used a supercell approach where each lattice vector of the rhombohedral unit cell was expanded three times, i.e. 3 $\times$ 3 $\times$ 3 supercell containing 432 ions in total. According to^35^, the 2^-^ charge state of the Ni vacancy is the most stable vacancy configuration in the wide range of Fermi level. Following that work, we use the same vacancy configuration (V$_{\text {Ni}^{2-}}$) in our calculations. Figure 5a shows the lattice distortions along the [100] and [110] crystallographic directions induced by a single nickel vacancy V$_{\text {Ni}^{2-}}$. Deformation along {111} directions are not shown due to the comparably small size. The vacancy induces an outward shift of the oxygen atoms along {100} crystallographic directions: the nearest oxygen atoms move away from the defect site by 0.14 Å. At the same time, along {110} directions the vacancy produces an inward shift: the nearest nickel atoms move towards the centre of the vacancy by 0.06 Å. This behaviour can be explained by electrostatic interaction between the charged vacancy and surrounding ions: negatively charged V$_{\text {Ni}^{2-}}$ repels oxygen anions and attracts nickel cations. The size of the distorted region is about 1.3 nm in diameter, which compares to the average size of nanodomains observed in STM.

In Fig. 5b we show the charge density difference induced by the V$_{\text {Ni}^{2-}}$ vacancy. It was calculated as a difference in charge density between the vacancy-containing supercell and the bulk supercell. However, in the bulk supercell we created the similar lattice distortions as in the vacancy-containing supercell. In Fig. 5b, one can see a distribution of defect states in real space. As expected, the vacancy site has a negative charge. The defect states exhibit a mixture of 3*d*–Ni and 2*p*–O states, though the latter dominates. The defect state is spread over a distance $\sim$ 4.7 Å, although slight excitations can be observed on oxygen atoms at the distance of 6.25 Å away from the vacancy centre. This result also agrees qualitatively with our STM and TEM observations of the nanodomains.

## Discussion

Park *et al.* have investigated point defects in NiO and the interaction between them^19^. Certain V$_{\text {Ni}}$–V$_{\text {Ni}}$ arrangements are strongly favoured over the non-interacting case. Specifically, nickel vacancies separated by around 8 Å along {001} directions is the favourable configuration. This is in agreement with asymmetric nanodomains presented in the HR STEM HAADF image (Fig. 4d), which are located in (001) plane and elongated in the [100] crystallographic direction of the NiO crystal.

**Figure 6:**
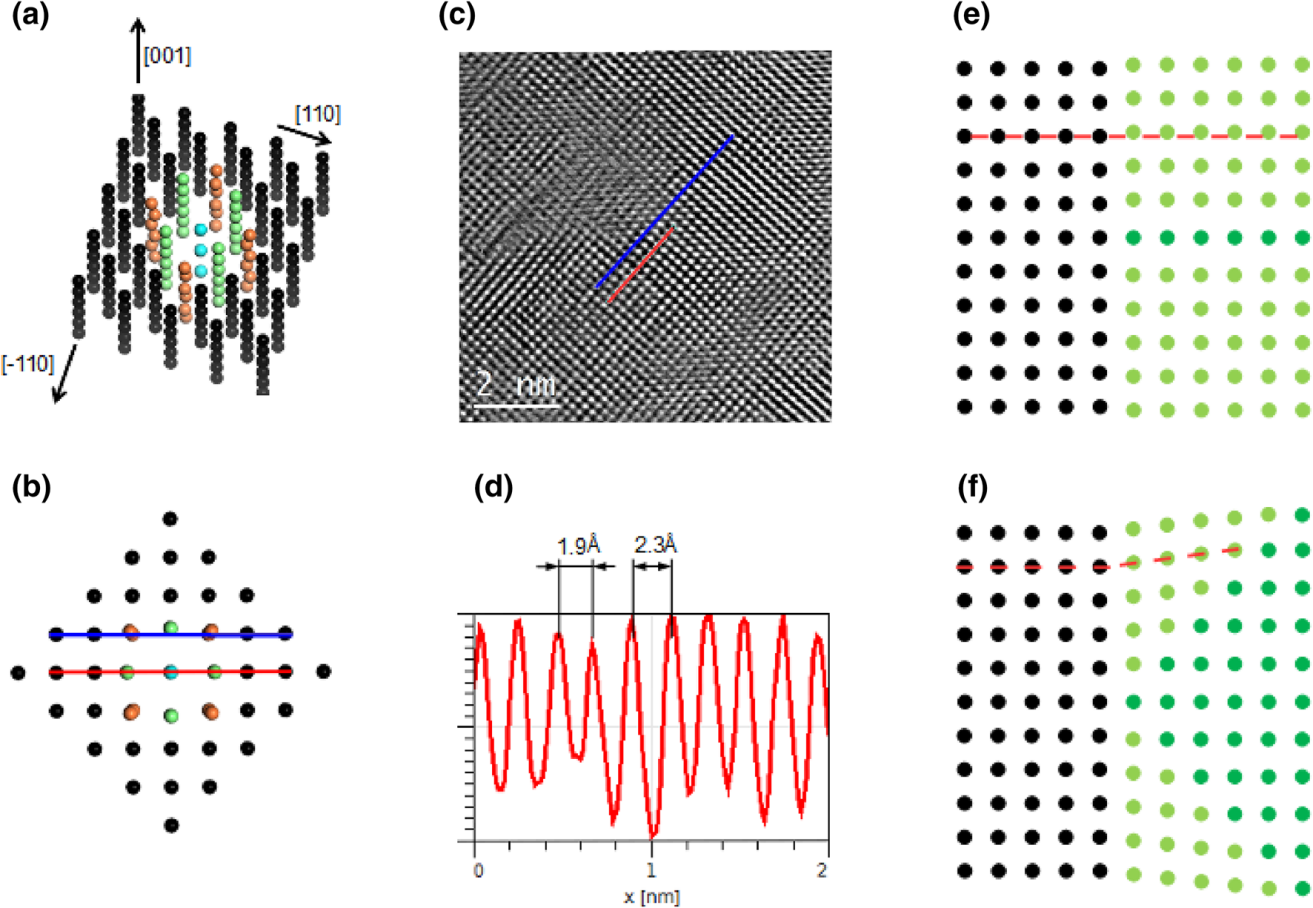
(**a**,**b**) Model containing a sequence of nickel vacancies along the [001] direction. Vacancies are within the blue column. The columns which experience significant relaxation due to the vacancies are depicted in green and orange. The relaxation—taken from the DFT calculation—is visualised in (**b**), which is a (001) plane. Red and blue lines in (**b**) illustrate the variation in the lattice constant and the bending of a lattice fringe induced by the vacancies. These predicted inhomogeneities are comparable to experiment; In (**c**) the blue line highlights an example of the bending of a lattice fringe, while (**d**) is the line profile corresponding to the red line in (**c**) and illustrates the variation in the lattice constant. (**e**) The dark green row represents a line (or plane out of the page) containing ordered nickel vacancies. The relaxation of adjacent rows (or planes) results in the decoherence of the light green and black regions, similar to an edge dislocation highlighted by the red-dashed line. (**f**) The triangular network of vacancies results in the light green and black regions being misorientated with respect to each other, highlighted by the red-dashed lines.

We now hypothesise how the range of observed inhomogeneities are correlated to the ordering of nickel vacancies. Figure 6a,b depicts a model containing several nickel vacancies separated by around 8 Å along the [001] direction. The atomic positions in the vicinity of the vacancies correspond to that of the fully relaxed DFT calculation. In Fig. 6a the atomic column containing vacancies is depicted in blue and the nearest and second-nearest parallel atomic columns are directed by green and orange, respectively. The relaxation in the vicinity of the vacancies is evident in (b), which is a top down view of (a). The red line in (b) illustrates the predicted variation of the lattice constant induced by the vacancies. This can be compared to the variation in the lattice constant observed by TEM; Fig. 6c shows the HR TEM image of the NiO crystal and (d) is a line profile corresponding to the red line in the TEM image. The local variation in the lattice constant is evident. The blue line in (b) illustrates how the vacancy network can give rise to the bending of a lattice fringe, which is comparable to the observed bending in TEM measurements, highlighted by the blue line in (c). Note the structure the TEM line profiles pass through are perfectly oriented along the [001] zone axis showing atomic resolved contrast. Therefore, the inhomogeneities in this area cannot be understood in terms of an isolated vacancy or randomly distributed vacancies. A one-dimensional row of vacancies along the [001] direction—predicted to be the favourable vacancy configuration—can give rise to the observed one-dimensional inhomogeneities. The nanodomain observed in STEM HAADF is suggested to be in the (001) plane of the NiO crystal. Within the nickel vacancy ordering picture, this domain can consist of two-dimensional network of vacancies within the (001) plane. Small domains, similar in size to those visualised in Fig. 4a, agglomerate due to interdomain interaction to form a larger features, which are visualised in AFM.

Figure 6e,f illustrates schematically how the ordered vacancies can produce lattice decoherence. The dark green in (e) illustrates a line (or plane perpendicular to the page) network of vacancies. The relaxation results in a shift of the adjacent atomic columns (or planes). As a result the light green region is shifted with respect to the black region. This is similar to an edge dislocation. If we consider a larger network of vacancies, such as the triangular network depicted in (f), one can visualise how the clustering of vacancies can result in the misorientation of regions (black and light green in schematic).

The presence of nanodomain structure has also been observed in the superconducting oxide YBCO with a orthorhombic structure^41^. In comparison to this work, the nanostructure inhomogeneity was attributed to nonstoichiometry.

## Conclusions

Different types of inhomogeneities in a range of submicron- to nanometer-scale are observed by a range of diffraction and microscopy techniques. The diffraction measurements illustrate the quality of the crystal but also a domain structure; XRD reveals domains of around 100 nm misoriented by several degrees, while LEED estimates domains of around 10 nm. AFM and STM image structures on the same length scale as XRD and LEED. In addition, TEM reveal structural inhomogeneity in the form of variation of the lattice constant, misorientation of domains and the bending of lattice planes. Finally, TEM reveals that nanodomains agglomerate into a larger submicron-scale structure.

The nature of the atomic-scale domain structure is studied using ab initio simulations. The distortion induced by a nickel vacancy is examined. This is the energetically favourable point defect and the reduced Ni/O ratio, observed by EELS and XPS, indicate its presence in the crystal. The DFT+U calculations reveal the spatial extent of the distorted area around the Ni vacancy is approximately 1.2 nm, close to that observed by TEM and STM. The defect state shows the same spatial distribution. The distance between overlapping defects is therefore 1.2 nm or less. Ordering of Ni vacancies is predicted to be favourable^19^. It is suggested that clustering of vacancies can give rise to formation of the range of inhomogeneities, the nature of which depends of the cluster size and structure. As such, the defects of different size, from atomic- to submicron-scale, are coupled.

## Methods

NiO and Ni$_{1-x}$Li$_x$O single crystals were grown by non-crucible FZM. Rods for FZM were prepared from high purity NiO and Li$_2$CO$_3$. The initial materials were weighed to the desired ratio, pressed into cylindrical rods and fired in air at 1100 $^{\circ }$C for 8 h. The sintered rods were float zone melted in ambient conditions at a growth rate of 10–12 mm/h. As a result both lithium doped ($\sim$ 1%) and undoped NiO single crystals, 35–40 mm in length and 3–4 mm in diameter, were obtained. Disk-shaped samples of 1 mm thickness and 4 mm diameter were prepared by cutting the cylindrical rods. The crystallographic orientation was controlled by back-Laue scattering, such that the surface normal of the disk was oriented along the [001] direction.

The AFM image was obtained with a Solver PROM from NT MDT. The images were obtained in tapping mode. The STM used in this work was a commercial slider-type STM from Createc. The images presented were obtained in constant-current mode at room temperature. The STM tip used was [001]-oriented single-crystalline tungsten, which was electrochemically etched in NaOH. The bias is applied to the sample with respect to the tip. LEED measurements have been performed in the same ultra-high vacuum chamber. In order to obtain a clean and ordered surface prior to in situ LEED and STM measurements, the surface was cleaned in situ by sputtering with argon ions for 1 h at each 3 kV, 2 kV, 1 kV and 0.5 kV in an argon partial pressure of 10$^{-5}$ mbar. After each iteration of sputtering the crystal was annealed in vacuum at 600 $^{\circ }$C for 1 h. The sample temperature was estimated with a K-type thermocouple. The crystal was then exposed to atmospheric conditions to recover the oxygen content and subsequently annealed in situ at 600 $^{\circ }$C in 20 min intervals until a sharp (1 $\times$ 1) LEED pattern was obtained.

XRD patterns have been measured with a Bruker D8 Discovery using a Cu-K$\alpha$ source. Crystal orientation was obtained using Bruker’s EVA program.

The samples for the TEM investigations were prepared on VERSA 3D HighVac dual beam facility (FEI) using the Ga ion beam. Regular FIB lamella preparation was carried out at 30 kV and followed by 2 kV final step to improve the surface quality. TEM investigations were performed on Titan 80-300 (FEI) transmission electron microscope equipped with GIF (Gatan) for the EELS study. The EEL spectra were collected at the convergence and collection angles of 14 and 15 mrad correspondingly, and with a spectral resolution of 0.2 eV/ch. For the EEL spectra quantification the background was fitted within an energy window of $\sim$ 50 eV with a power-law function. The sample thickness was less than the mean free path so the procedure of multiple inelastic scattering deconvolution was not performed. The intensity beyond the edge onset was integrated over a window of 70 eV to minimize the influence of the corresponding near-edge fine structure. The setup for HR STEM imaging was a 50 $\upmu$m C2 aperture, convergence angle of 10 mrad, camera length of 195 mm and collection angle 30–180 mrad.

First-principles electronic structure calculations of NiO were performed using the projector augmented wave (PAW) method^42^ implemented in Vienna ab initio simulation package (VASP)^43–45^. The exchange-correlation effects were treated using the spin-polarised generalised gradient approximation^46^ in combination with the Hubbard Coulomb term (GGA + U) according to the Dudarev scheme^37^. Below the Néel temperature, NiO exhibits type-II antiferromagnetic order. In calculations the magnetic properties were accounted for within the collinear picture. The magnitude and orientations of the collinear local moments were calculated self-consistently. The cut-off energy for plane waves was set to 500 eV. The integration over the irreducible part of Brillouin zone has been carried out using the Monkhorst–Pack method^47^ on grids of $6\,\times \,6\,\times \,6$ k-points. The convergence criterion for electronic subsystem was $10^{-4}\,\hbox {eV}$ for subsequent iterations. The relaxation of atomic positions was realised by calculating the Hellman–Feynman forces^48,49^ and stress tensor using the conjugated gradient method. Relaxation was stopped when the forces became on the order of $5\,\times \,10^{-3}\,\hbox {eV}$/Å.

## Supplementary Information

Supplementary Information.
