# Supplementary material for: Nanodomain structure of single crystalline nickel oxide
Source: Sci Rep. 2021 Feb 10;11:3496. doi: 10.1038/s41598-021-82070-1 (PMC7875979; doi:10.1038/s41598-021-82070-1)
Supplement: Supplementary file 1 — Supplementary Information. [file 41598_2021_82070_MOESM1_ESM.pdf]

Supplementary Information -
Nanodomain structure of single crystalline nickel oxide

B. Walls, A. A. Mazilkin, B. O. Mukhamedov, A. Ionov, I. A. Smirnova, A. V. Ponomareva, K. Fleischer, N. A. Kozlovskaya, D. A. Shulyatev, I. A. Abrikosov, I. V. Shvets, S. I. Bozhko

1 X-ray diffraction of the Li doped NiO crystal

Below is the X-ray diffraction (XRD) reciprocal space map (RSM) of both the doped and undoped sample. The measurement of the undoped crystal - which is included in the main text - is included here for comparison to the Li doped crystal.

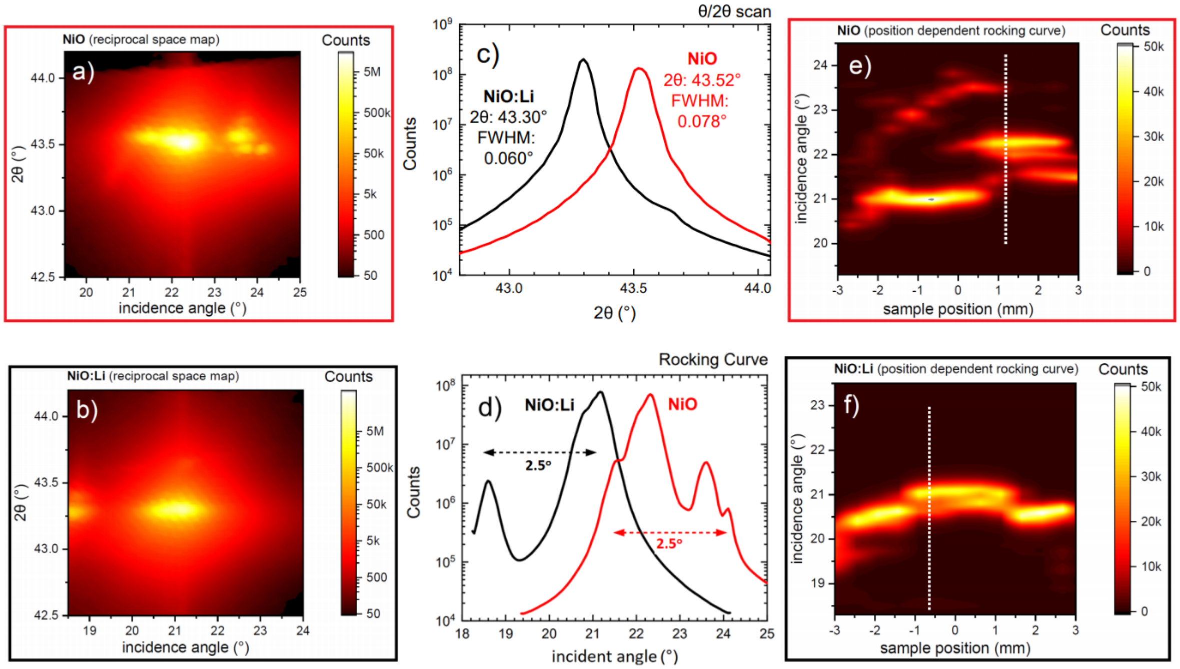

Supplementary Fig. S1 Reciprocal space map shown in terms of incident angle and 2θ angle for (a) undoped and (b) Li doped NiO. (c) shows $\theta/2\theta$ scan for both samples obtained by summing all the columns of each reciprocal space map. The doped sample has a significantly sharper diffraction peak indicating overall better crystallinity. (d) shows the corresponding rocking curve derived from summing all the rows of the reciprocal space map. The Li doped sample is more homogeneous showing a sharper rocking curve with less substructure compared to the NiO. The indicated angular interval shows that there are misoriented areas within the probed sample volume. (e) and (f) show rocking curves as a function of sample position. The sample was moved by 0.5 mm per step. Dotted lines indicate the region of the sample where (a)-(d) have been taken.

Reciprocal space maps of the NiO and the Li doped NiO crystals are depicted in (a) and (b), respectively. (c) shows the $\theta/2\theta$ scan for both samples. The lattice constant of the doped crystal is greater than that of the undoped crystal. The overall crystallinity is improved upon doping with a sharper diffraction pattern. Using the Scherrer equation we can estimate the average coherent domain size to be 150±1 nm and 115±2 nm for the $Ni_{1-x}Li_xO$ and NiO crystals, respectively.

The corresponding rocking curves are presented in (d). We observe defined fine structure with two distinct reflexes separated by a few degrees in both cases. Taking rocking curves at several areas of the sample in 0.5 mm steps (see (e) and (f)) reveals that the tilt of the domains is maintained over the probed region. The $Ni_{1-x}Li_xO$ has a macroscopic more homogeneous structure with the misorientation being consistent over the probed region.

The domain structure of the doped crystal is qualitatively comparable to that of the undoped crystal; both exhibit a distinct fine structure with the misalignment of domains maintained over the probed region.

2 Elemental characterisation

Electron energy loss spectroscopy (EELS) estimation of the NiO elemental composition is depicted below. It demonstrates a small deficiency of Ni cations. EELS measurements were performed on five different samples on ten different sample areas each on average. For each measurement we observed a Ni deficiency. The average Ni content was 48%.

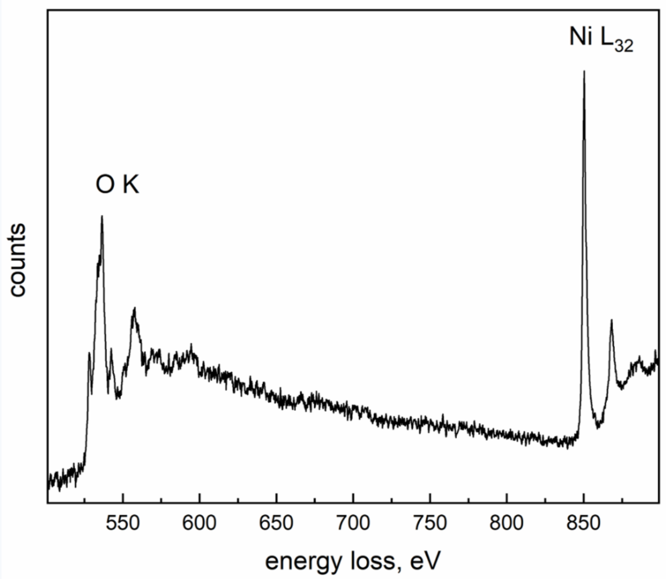

Supplementary Fig. S2 Core loss EELS of the NiO single crystal. The spectrum contains O K and Ni L_{32} edges. Quantification of the elemental composition (48/52%) demonstrates a small deficiency of Ni cations.

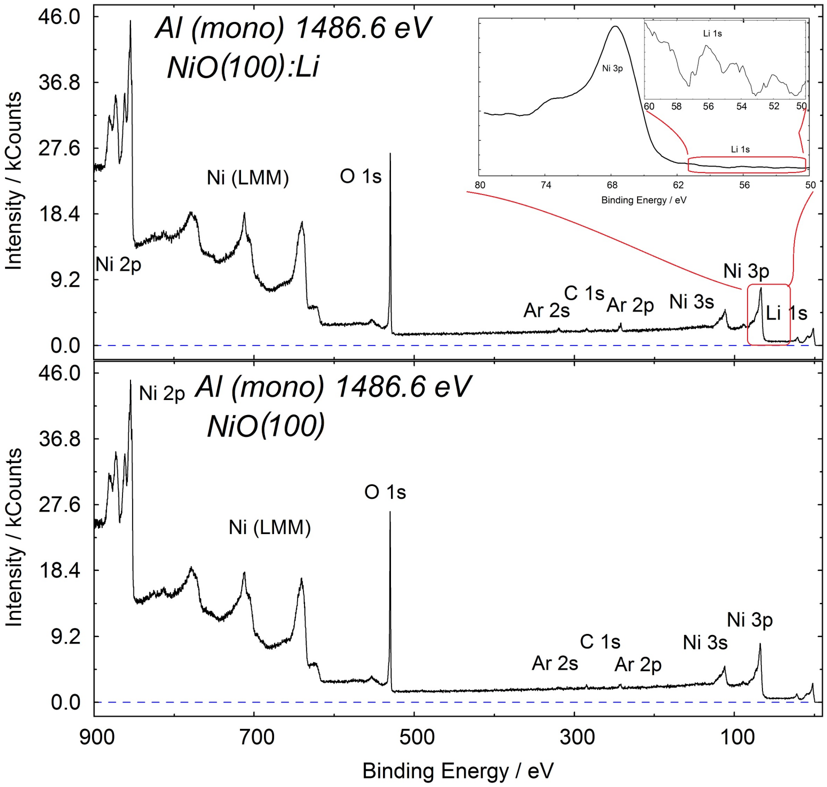

Supplementary Fig. S3 X-ray photoelectron spectra of the Li doped (top) and undoped (bottom) NiO crystal.

X-ray photoelectron spectroscopy measurements of both the doped and undoped crystals are presented above. In both cases the spectra shows the presence of Ni and O peaks with very small peaks attributed to argon and carbon. In the case of the Li doped crystal we also observe small Li peaks, which are visualised in the inset of the top image. The atomic ratio of the Ni to O was estimated to be 48.5:51.5 and 48:52 in case case of the doped and undoped crystal, respectively. In the doped crystal the Li content was estimated to be 0.4±0.1%. The estimation of the Ni to O ratio was obtained by analysing the Ni 2p$^{3/2}$ and O 1s areas[1]. The Shirley method was employed for the background subtraction.

References

1. Vardimon, R., Klionsky, M. & Tal, O. Indication of complete spin filtering in atomic-scale nickel oxide. *Nano Lett.* **15**, 3894–3898, DOI: 10.1021/acs.nanolett.5b00729 (2015).
